# Langerhans Cell Histiocytosis of the Orbit Presenting as Periorbital Cellulitis

**DOI:** 10.7759/cureus.32656

**Published:** 2022-12-18

**Authors:** Mohammad Anwar, Mohamed Eltayef

**Affiliations:** 1 Rheumatology, Hampshire Hospitals NHS Foundation Trust, Basingstoke, GBR; 2 Ophthalmology, Hampshire Hospitals NHS Foundation Trust, Basingstoke, GBR

**Keywords:** vinblastine, ocular proptosis, periorbital edema, orbital, langerhans cell histiocytosis(lch)

## Abstract

Langerhans cell histiocytosis (LCH) is a rare disease of the reticuloendothelial system. It is characterized by misguided differentiation of myeloid dendritic cell precursors. LCH most commonly presents in childhood and the clinical presentation is dependent on the site and extent of organ involvement. We report a case of orbital Langerhans cell histiocytosis in a 12-year-old boy who presented with left periorbital cellulitis with subsequent proptosis. He later underwent a successful left orbital-frontal-temporal craniotomy and is currently undergoing postoperative chemotherapy.

## Introduction

Langerhans cell histiocytosis (LCH) is a rare inflammatory/neoplastic condition of dendritic cells. It is diagnosed on the basis of histology and has positive immunophenotypic markers for CD1a, langerin (CD207), S100, and cytoplasmic Birbeck granules [1]. While initially based on the similarities in immunophenotypic markers, LCH was thought to arise from skin Langerhans cells which are antigen-presenting cells in the epidermis. It is now thought that LCH occurs as a consequence of a misguided differentiation program of myeloid dendritic cell precursors due to the presence of BRAF V600E mutations [1]. Patients with BRAF V600E mutations have a significantly higher risk for relapses [2]. The incidence of LCH is around 5.4 cases per million people per year and it has a slight male predominance [1,3].

LCH can present with single-system or multi-system involvement. Due to the rare incidence of LCH and incomplete understanding of its pathogenesis, treatment protocols are not yet standardized. Treatment of LCH is typically a combination of prednisone and vinblastine in multisystem LCH as per LCH-III trials. Orbital lesions with an intracranial extension are regarded as CNS-risk lesions and need systemic chemotherapy. Curettage is usually reserved for single-system unifocal bone involvement (isolated bone lesions).

LCH-IV trials are currently underway to tailor treatments based on features at presentation and on response to treatment to various modalities/drugs including prednisone, vinblastine, mercaptopurine, indomethacin, methotrexate, cytosine arabinoside, 2-chlorodeoxyadenosine, hematopoietic stem cell transplantation and intravenous immunoglobulins.

Orbital cellulitis is an infection of the tissues posterior to the orbital septum. The most common cause of orbital cellulitis is paranasal sinuses. Other sources include spread from adjacent ocular structures, traumatic injury, ophthalmic surgery, dental infection and upper respiratory tract infection. The differential diagnoses of orbital cellulitis include neoplasia, trauma (retrobulbar haemorrhage/orbital emphysema), thyroid eye disease, non-specific orbital inflammation (orbital pseudotumor) or autoimmune diseases (sarcoidosis or granulomatosis with polyangiitis).

## Case presentation

A 12-year-old boy presented to the emergency department with left eye swelling for one week with a one-day history of headache. He had been started by his general practitioner (GP) on oral clarithromycin during the previous week for suspected left periorbital cellulitis. The patient did not have any fever/neck pain/rash/previous ENT or dental problems. His past medical history included hay fever and a chickenpox infection eight years ago.

**Figure 1:**
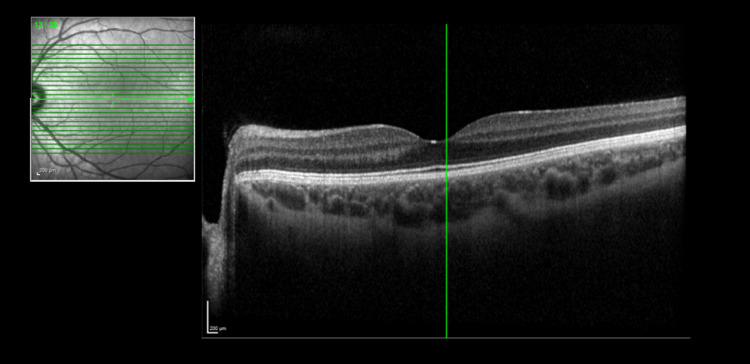
Optical coherence tomography (OCT) scan of the left eye

**Figure 2:**
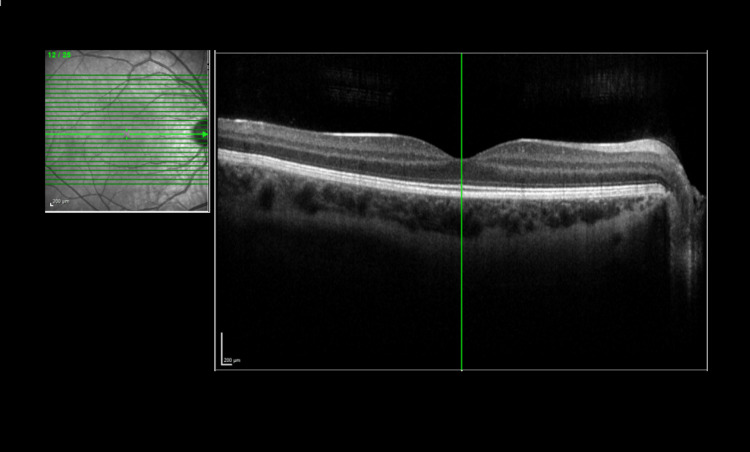
Optical coherence tomography (OCT) scan of the right eye

On examination in the emergency department, it was noted he had some left periorbital swelling and tenderness at the left supra-orbital and frontal sinus regions. He had a full range of left eye movement with minimal pain. Pupils were equal and reactive in both eyes and visual acuity was normal in both eyes. He was referred to the ophthalmology team who examined him the next day. External examination showed mild periorbital oedema around the left eye. He had 6/5 visual acuity in both eyes. Fundus examination was unremarkable. His macula and optic disc were normal on OCT (optical coherence tomography) scan for both eyes. The OCT scan for the left eye (Figure 1) and the right eye (Figure 2) are shown below.

It was decided to continue with oral clarithromycin and to review him in the clinic after three days. He was reviewed in the clinic after three days and it was then noted that he had developed mild left eye proptosis.

**Figure 3:**
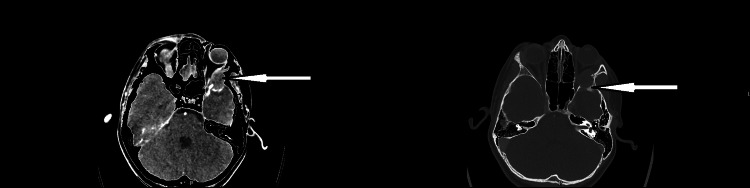
CT head axial section with soft tissue window (left side) and bone window (right side). The soft tissue window (left side) shows a collection at the left orbital wall, and the bone window (right side) shows bony destruction of the greater wing of the sphenoid bone.

An urgent CT scan of the head with orbit was done which reported a 22 x 20 x 15 mm peripherally enhancing collection at the left orbital wall with focal destruction of the underlying bone, effacement of the left lateroconal structures and 7 mm abutment through into the anterior middle cranial fossa and effacement of the temporal pole. The conclusion was suggestive of a collection focused on the left orbital wall with intracranial extension. A side-by-side comparison of the soft tissue window (left side) and bone window (right side) of the axial section of the CT head is shown below (Figure 3).

He was admitted under the paediatric team and started on IV ceftriaxone and metronidazole and was referred to neurosurgery at a tertiary centre which recommended an MRI of the head region. His admission blood work-up showed slightly elevated C-reactive protein of 11 mg/L (reference range from 0-5 mg/L), and elevated prothrombin time of 14.8 seconds (reference range from 12.5-14.4 seconds). The remaining blood tests were unremarkable including an ESR (erythrocyte sedimentation rate) of 6mm/hr (reference range from 1-10 mm/hr), white blood cell count 8.7 x109/L (reference range from 4.5-13 x 109/L), and creatinine of 56 umol/L (reference range from 36-67 umol/L). Blood cultures were also taken and were later reported as negative. Blood test results are summarised in Table 1.

**Table 1:** Summary of blood test results

Blood test	Result	Reference
C-reactive protein	11 mg/L	0-5 mg/L
Erythrocyte sedimentation rate	6 mm/hr	1-10 mm/hr
Haemoglobin	138 g/L	110-145 g/L
White blood cell count	8.7 x 10^9^/L	4.5-13 x 10^9^/L
Platelet count	348 x 10^9^/L	150-500 x 10^9^/L
Neutrophil count	5.48 x 10^9^/L	2-6 x 10^9^/L
Creatinine	56 umol/L	36-67 umol/L
Sodium	136 mmol/L	133-146 mmol/L
Potassium	4.4 mmol/L	3.5-5 mmol/L
Alkaline phosphatase	397 U/L	60-425 U/L
Prothrombin time	14.8 seconds	12.5 - 14.4 seconds
Activated partial thromboplastin time	32.8 seconds	24.5 - 37.1 seconds
Blood cultures	Negative	-

**Figure 4:**
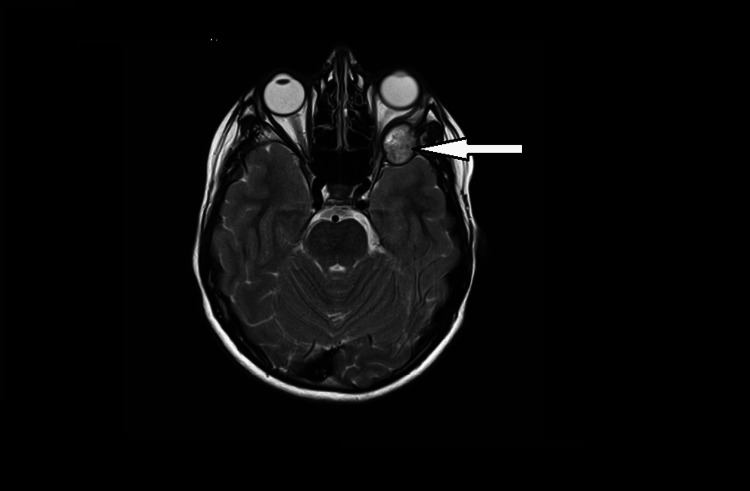
Axial MRI T2-weighted MRI imaging of the head The MRI shows a 2 cm lesion marked with an arrow.

An MRI head with gadolinium contrast was performed and reported a 2 cm enhancing mass with lytic destruction of the greater wing of the sphenoid with impingement into the retro-orbital fat and the anterior skull base with effacement of the left anterior temporal lobe. An axial T2 weighted image showing a 2 cm lesion is shown in Figure 4.

**Figure 5:**
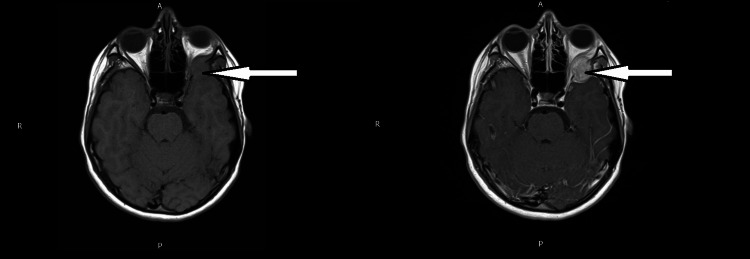
MRI imaging of the head in axial view with T1 pre-gadolinium (left side) and post-gadolinium contrast (right side) Enhancement of hyperintense lytic lesion with gadolinium contrast on the right side as compared to the pre-contrast image on the left side.

An axial view of the MRI of the head with T1 pre-gadolinium (left side) and post-gadolinium contrast (right side) is shown in Figure 5, demonstrating an enhancing hyperintense lytic lesion with gadolinium contrast as compared to the pre-contrast hypointense lesion.

MRI scan results and blood tests were discussed with the neurosurgery team who advised that IV antibiotics were to be stopped. The patient was then transferred to a tertiary centre for consideration of biopsy under the neurosurgical team. Further tests were performed including a chest X-ray, abdominal ultrasound and a skeletal survey which were all unremarkable. Plasma osmolality was also found to be within the normal range. Viral serology showed IgG +ve for varicella and measles. Epstein-Barr virus (EBV) and cytomegalovirus (CMV) serology were negative. He was discussed in the skull base multidisciplinary team (MDT) and it was decided to perform left orbital-frontal-temporal craniotomy for excision of the tumour in the left lateral wall of the orbit followed by orbital reconstruction with plates.

The patient had a successful surgery and was then discharged home with the outpatient paediatric ophthalmology clinic for follow-up and showed excellent postoperative visual acuity in the left eye 6/6. He had a slight restriction in left eye abduction (controlled by the left lateral rectus muscle) and an element of resolving proptosis of the left eye.

Histopathology of the excised mass reported a dense infiltrate of inflammatory cells containing a population of histiocyte-like cells with neutrophils, lymphocytes, eosinophils and occasional multinucleated cells. The histiocytic cells were labelled positively with langerin, CD1a and S100 and were negative for CD68. In conclusion, the report was suggestive that the appearances were consistent with that of Langerhans cell histiocytosis.

His case was discussed by the neuro-oncology multidisciplinary team (MDT) and the diagnosis was confirmed as Langerhans cell histiocytosis with single system (bony) disease with single site (left orbit) involvement. In view of the anatomical site of the disease (orbit), it was considered a central nervous system risk site and it was therefore decided to start him on a chemotherapy regimen of vinblastine and prednisolone. The patient is currently undergoing chemotherapy.

## Discussion

Langerhans cell histiocytosis was formerly known as histiocytosis X. Histologically, LCH was subdivided into three categories: eosinophilic granuloma (causing solitary/multifocal bony abnormalities), Hand-Schuller-Christian disease (a triad of calvarial lesions, diabetes insipidus and exophthalmos), and life-threatening Letterer-Siwe disease (multi-system disease) [4]. Eosinophilic granuloma causes 60% to 70% of all cases of LCH [5]. Clinically, LCH is classified into three distinct forms: single-system single-site (SS-s), single-system multi-site (SS-m), and multi-system type (MS) [6]. Skeletal involvement is one the most common features of LCH [7] and presents as lytic lesions. Bony destruction results from increased production of cytokines including IL-1 (which impairs collagen formation and is also a potent osteoclast activating factor) and PGE2 (which indirectly enhances osteoclast formation) [8]. Orbital LCH is usually associated with a lytic lesion of the orbital wall. Differential diagnosis of an orbital lytic bony lesion may include leiomyoma, myofibroma, solitary fibrous tumours, primary intraosseous hemangiomas, hemangiopericytoma, rhabdomyosarcoma, chondrosarcoma, myelogenous leukaemia, neuroblastoma and osteosarcoma. [8]

Orbital LCH usually presents as upper eyelid swelling which can be associated with exophthalmos or proptosis. Orbital LCH can be mistaken for peri-orbital cellulitis as the growth of LCH causes an inflammatory response. Orbital LCH may also result in cranial nerve palsies, papilledema and secondary optic atrophy. [9]

Due to the rare incidence of LCH and incomplete understanding of its pathogenesis, treatment protocols are not yet standardized. Treatment of LCH is typically a combination of prednisone and vinblastine in multisystem LCH as per LCH-III trials. Orbital lesions with an intracranial extension are regarded as CNS-Risk lesions and need systemic chemotherapy [10]. Curettage is usually reserved for single-system unifocal bone involvement (isolated bone lesions). LCH-IV trials are currently underway to tailor treatments based on features at presentation and on response to treatment to various modalities/drugs including prednisone, vinblastine, mercaptopurine, indomethacin, methotrexate, cytosine arabinoside, 2-chlorodeoxyadenosine, hematopoietic stem cell transplantation and intravenous immunoglobulins.

LCH is associated with other malignancies including acute lymphoblastic leukaemia or lymphoma which may occur either before or after the diagnosis of LCH [11].

## Conclusions

LCH is a rare disease which can present with a spectrum of clinical presentations based on specific anatomical site involvement. Orbital involvement is a rare manifestation of LCH and causes lytic bony lesions, cranial nerve palsies, papilledema and secondary optic atrophy. Treatment of multi-system LCH is typically a combination of prednisone and vinblastine. Orbital lesions with an intracranial extension are regarded as CNS-risk lesions and need systemic chemotherapy. LCH-IV trials are currently underway to tailor treatments based on features at presentation and on response to treatment to various modalities/drugs.
